# Effect of Graphene Nanosheets Content on Microstructure and Mechanical Properties of Titanium Matrix Composite Produced by Cold Pressing and Sintering

**DOI:** 10.3390/nano8121024

**Published:** 2018-12-08

**Authors:** Milad Haghighi, Mohammad Hossein Shaeri, Arman Sedghi, Faramarz Djavanroodi

**Affiliations:** 1 Department of Materials Science and Engineering, Imam Khomeini International University (IKIU), Qazvin 3414916818, Iran; miladhaghighi@outlook.com (M.H.); sedghi@eng.ikiu.ac.ir (A.S.); 2 Mechanical Engineering Department, Prince Mohammad Bin Fahd University, Al Khobar 31952, Saudi Arabia; f.djavanroodi@imperial.ac.uk; 3 Department of Mechanical Engineering, Imperial Collage London, London SW7, UK

**Keywords:** titanium matrix composite, mechanical properties, microstructure, graphene nanosheets

## Abstract

The effect of graphene nanosheet (GNS) reinforcement on the microstructure and mechanical properties of the titanium matrix composite has been discussed. For this purpose, composites with various GNS contents were prepared by cold pressing and sintering at various time periods. Density calculation by Archimedes’ principle revealed that Ti/GNSs composites with reasonable high density (more than 99.5% of theoretical density) were produced after sintering for 5 h. Microstructural analysis by X-ray diffraction (XRD) and a field emission scanning electron microscope (FESEM) showed that TiC particles were formed in the matrix during the sintering process as a result of a titanium reaction with carbon. Higher GNS content as well as sintering time resulted in an increase in TiC particle size and volume fraction. Microhardness and shear punch tests demonstrated considerable improvement of the specimens’ mechanical properties with the increment of sintering time and GNS content up to 1 wt. %. The microhardness and shear strength of 1 wt. % GNS composites were enhanced from 316 HV and 610 MPa to 613 HV and 754 MPa, respectively, when composites sintered for 5 h. It is worth mentioning that the formation of the agglomerates of unreacted GNSs in 1.5 wt. % GNS composites resulted in a dramatic decrease in mechanical properties.

## 1. Introduction

Metal matrix composites (MMCs), such as aluminum matrix composites (AMCs) and titanium matrix composites (TMCs), have the potential to improve the physical and mechanical characteristics of metallic materials, such as their modulus and strength [1,2]. Titanium matrix composites (TMCs) are an ideal choice for engineering applications, such as structural materials at high temperatures, aerospace, armor, and medical and chemical fields, owing to their excellent specific modulus and strength, high corrosion resistance, elevated temperature resistance, and low density [3,4,5,6]. In addition to selecting a suitable matrix, the selection of the best possible reinforcement is also important for obtaining the desired properties in the production of a composite. In other words, the performance of the MMCs extremely depends on the properties of selected reinforcements, such as Young’s modulus, strength, size, morphology, etc. [1].

A large number of TMCs have been produced with ceramic reinforcements, such as TiB [7], Al_2_O_3_ [8], TiN [9], SiC [10], and TiC [11]. Although these reinforcements improve the mechanical properties and have suitable compatibility, their densities are higher than that of titanium [12]. In addition, the elastic modulus, fracture toughness, and specific surface area of ceramic reinforcements are less than those of carbon nanostructures, such as carbon nanotube (CNT) and graphene. Thus, it is clear that carbon structures can improve TMCs’ properties to a greater degree. In recent years, carbon nanotube (CNT) and graphene have been extensively considered for their superior qualities as excellent nanofillers [13].

Graphene is a single atomic layer of sp2-hybridized carbon atoms packed densely in a honeycomb lattice that has superior properties, such as remarkable electrical and thermal conductivity (due to high electron mobility) and extreme mechanical strength. Moreover, the high specific surface area and modulus of graphene have been reported in theoretical and experimental studies [14,15,16,17,18,19,20]. Hereby, graphene has been used in some metal matrix composites, such as aluminum [21,22] and magnesium [23,24] matrix composites, as a suitable reinforcement. Graphene has a higher specific surface area compared to CNT and, consequently, the contact area of graphene with the matrix is reasonably larger than that of CNT at the same mass fraction. Therefore, it is expected that the transmission of stress through their stronger interfacial bonding is notably easier. Additionally, graphene could create a balance between strength and ductility in the composites. For all these reasons, graphene can be a suitable candidate to use as a TMC’s reinforcement. However, it should be noted that graphene reacts with titanium at high temperature and TiC particles are formed. A continuation of this reaction at a high temperature and, consequently, the incorporation of the particles as the hard reinforcements in the titanium matrix may destroy some effects of nanographenes [25,26,27].

Many investigations have been focused on the manufacturing of CNT-reinforced titanium-based composites [13,28,29,30], but fewer studies and experiments have been conducted on the mechanical properties and microstructure of TMCs reinforced with graphene. Cao et al. [31] fabricated titanium (Ti–6Al–4V)/graphene nanoflake (GNF) composites via hot isostatic pressing (HIP) followed by isothermal forging and reported that both the yield and ultimate tensile strengths were increased with the addition of GNFs to the matrix, without the loss of ductility. Additionally, Mu et al. [32] produced a titanium matrix composite reinforced with graphene nanoplates (GNPs) using spark plasma sintering (SPS) and subsequent hot-rolling. Similar to GNFs, the addition of GNPs to titanium leads to a significant increment in mechanical properties. Due to the limited research on the Ti/graphene composites, fabricating this composite using a simple method, such as cold press/sintering, and studying the parameters of the fabrication process, such as the sintering time, can be worthy.

In current research, the powder metallurgy (PM) process, including the three steps of dispersion, cold pressing, and sintering, was utilized to produce titanium matrix composites with various weight percentage of graphene nanosheets (GNSs) and different sintering times. Following the samples’ fabrication, the effects of the volume fraction of reinforcements and time periods of sintering on the microstructure and mechanical properties of TMC were studied. The density of the composites was measured by Archimedes’ method. The phase composition of the samples was determined by x-ray diffraction (XRD) and energy-dispersive detector (EDS) and microstructures of the composites were investigated using field emission scanning electron microscopes (FESEM). The hardness and shear punch test were also employed to measure the mechanical properties of composites.

## 2. Materials and Methods

**Figure 1:**
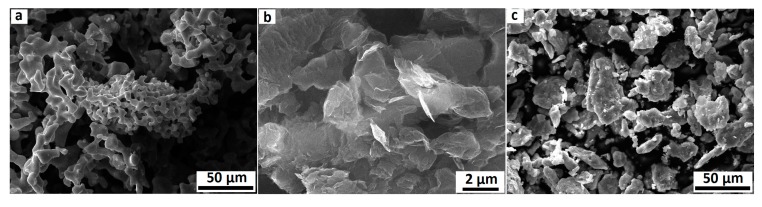
SEM image of (**a**) pure titanium powder before ball milling, (**b**) graphene nanosheets (GNSs) before ball milling, and (**c**) mixed powders after ball milling.

Titanium (purity >99.8%) with a maximum particle size of 40 μm from JSC POLEMA (Tula, Russia) and graphene nanosheets with a width size less than 5 μm and the maximum of 2 to 4 layers with a surface area of 350 m^2^/g were used as raw materials in this work. Figure 1a,b shows the SEM images of pure titanium powder and GNSs. As can be seen, GNSs represent a high aspect ratio and two-dimensional layers.

The manufacturing process of titanium/graphene composites generally consists of the three steps of dispersion, cold pressing, and sintering. First, to evaluate the effect of GNS content on the microstructure and mechanical properties, 0.5, 1 and 1.5 weight percent (wt. %) of GNSs were added to the pure titanium powder. Then, ball milling (BM) was used for mixing GNSs and pure titanium powders as one of the effective methods of graphene dispersion in the matrix [33]. The powders were mixed in stainless steel cups containing alumina balls with a weight ratio of ball to powder 10:1 for 3 h at a speed of 100 rpm in an argon atmosphere. Figure 1c shows the partial crushing of GNSs during the milling, which leads to reasonable attachment of titanium powders to GNSs. As can be seen in Figure 1, the size of the titanium particles was reduced from 40 to 20 μm after the ball milling. In the second step, the mixed powders were poured into a cylindrical steel die with an internal diameter of 12 mm and were subsequently pressed by a unidirectional press machine under pressure of 850 MPa for 10 min at room temperature. Due to the significance of the friction parameter on pressure transfer from die to powders, “Moly Coat 1000 Paste” lubricant was used between the die walls and the powders. After cold pressing, sintering in an argon-protected vacuum furnace at 1273 K for 1, 3, and 5 h was carried out to study the effect of sintering time on mechanical properties and the microstructure of composites. The rate of heating to the sintering temperature was 5 K/min and the samples were furnace cooled.

Densities of pure Ti and Ti–GNS composites were calculated using Archimedes’ principle. A digital density meter with a precision of 0.01 mg was employed to measure the density. At first, the samples of pure Ti and Ti/GNSs composites were cleaned. Then, the samples were weighed in air and distilled water and, subsequently, the density was measured using Archimedes’ method. Theoretical densities were also computed using the rule of mixtures by taking theoretical densities of 4.506 g/cm^3^ for titanium and 2.00 g/cm^3^ for GNSs.

X-ray diffraction analysis (PW1730 diffractometer, Philips, Kassel, Germany) was conducted to characterize the second phases in the composites. X-ray CuKα was used at a wavelength of 1.5404 Ả with diffraction angles (2θ) ranging from 20° to 90°. The microstructure and surface morphology of the composites were also studied using a TESCAN-MIRA3 field emission scanning electron microscope (TESCAN BRNO, Kohoutovice, Czech Republic) equipped with EDS. To prepare the samples for the SEM, samples were first divided into two sections in the longitudinal direction. Then, grinding and polishing operations were performed with standard methods of preparation. The solution of 100 mL distilled water, 5 mL hydrogen peroxide (H_2_O_2_), and 2 mL hydrofluoric acid (HF) was employed for etching the specimens’ surface.

**Figure 2:**
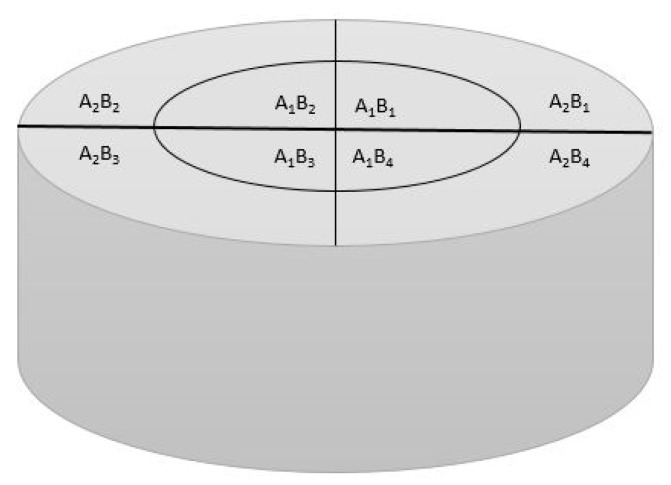
Schematic of partitioned surface of the samples used for microhardness measurement.

The hardness of the samples was measured by a Vickers microhardness test using the HVS-1000A instrument (Laizhou Lyric Testing Equipment Co., Shandong, China) with a load of 500 g and dwell time of 15 s, in accordance with ASTM E-384 standard (ASTM: American Society for Testing and Materials). According to Figure 2, to increase the accuracy of the measurements and check the uniformity of the surface hardness, the surface of the specimens’ cross section was divided into eight sections, and the microhardness of each section was measured.

The shear punch test is an appropriate choice for measuring the mechanical properties of small-dimensional or thin-sectioned specimens. Thin plate samples with a thickness of 0.7 mm were prepared in a transverse direction. These sheets were placed in a shear punch die with a punch and die inside diameters of 6.2 mm of 6.25 mm, respectively. Detailed information of shear punch equipment utilized in this study is represented in our previous papers [34,35]. All the shear punch tests were performed at room temperature using a Zwick/Roell Z100 Universal Testing machine (ZwickRoell GmbH & Co., Ulm, Germany). No lubrication between sheet and die was used. The applied force was measured in terms of punch displacement, and shear stress was calculated in MPa using the following equation [36,37]:

(1) $$\tau =\frac{P}{\pi \mathrm{dt}}\;,$$

where P is the punch force in newton, t is the sample thickness, and d is the average diameter of the punch and die in mm. The shear punch test curves were obtained by plotting shear stress vs. normal displacement. Normal displacement was obtained via the following equation [36,37]:

(2) $$d=\frac{h}{t},$$

where h is the punch displacement in mm. The test was repeated 3 times and the average magnitudes of the shear yield and ultimate shear strengths were reported.

## 3. Results and Discussion

### 3.1. Density

Theoretical and experimental densities of pure Ti and Ti/GNSs composites calculated by Archimedes’ principle are listed in Table 1. 

According to Table 1, by increasing the sintering time at 1273 K from 1 to 5 h, the density of the samples increased in composites containing the same weight percentage of GNSs. The main reasons lie in the elimination of porosities as a result of increasing the diffusion of cavities at high temperatures and their reaching out to the composite surface, and also producing higher amounts of TiC, which possesses a reasonably higher density compared to that of GNSs (ρ_GNS_ = 2.00 g/cm^3^, ρ_TiC_ = 4.93 g/cm^3^). On the other hand, by increasing the weight percentage of the GNSs, the density of composites decreased slightly, irrespective of sintering time. The reduction of density by increasing the GNS content in composites was due to the relatively low density of GNSs, as well as the negative effect of unreacted GNSs on the densification of the samples [38].

### 3.2. XRD Analysis

**Figure 3:**
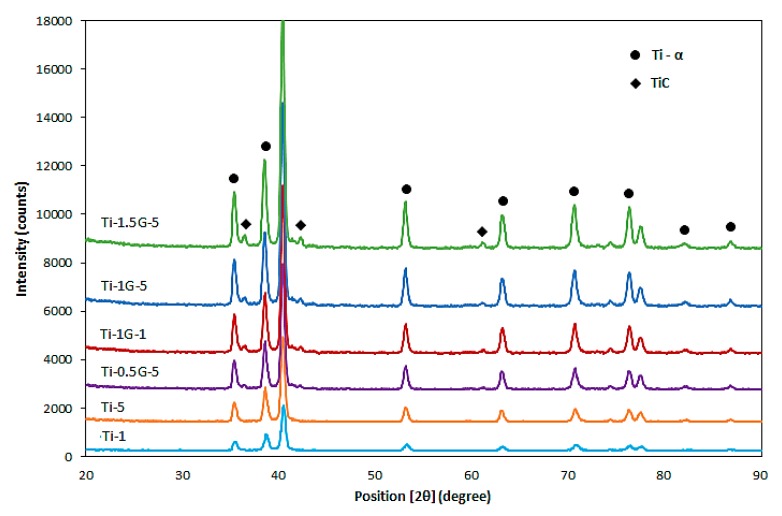
XRD patterns of Ti/GNS composites fabricated at various conditions.

XRD patterns of pure titanium and Ti/GNSs composites are shown in Figure 3. According to reference JCPDS NO: 44-1294, the main phase in pure titanium is α-Ti. It is clear that the pure titanium composite sintered for 5 h has a sharper peak than the composite sintered for 1 h, which indicates a more homogeneous structure with a higher density. The XRD patterns of composites with different weight percentages of GNS reinforcement sintered for 5 h reveal that adding the GNSs to the titanium matrix resulted in the appearance of the TiC peaks, indicating the formation of TiC during sintering at 1273 K (according to reference: JCPDS NO: 06-0614). These observations demonstrate that titanium has reacted with GNSs during sintering and TiC particles have been created. In accordance with thermodynamic theories, the Gibbs free energy for the reaction between titanium and GNSs at 1273 K was calculated to be about −181 kJ/mol for the solid state, showing the spontaneous nature of in-situ formation of TiC during sintering [25]. It is clear that with increasing the weight percentage of GNSs, the relative intensity and width of the TiC peaks increased. In other words, increasing the amount of GNSs in the Ti/GNSs composite caused an enhancement in the volume fraction and particle size of TiC particles. Comparing the patterns of the samples containing 1 wt. % of GNSs sintered for 1 and 5 h in Figure 3 indicates that the TiC peaks are somewhat sharper and more intense in composite sintered for 5 h in comparison with those sintered for 1 h. Thus, it can be deduced that increasing the sintering time leads to encouraging the formation of TiC particles and also increases the grain size of the TiC particles as a result of the intense thermal movement of titanium and carbon atoms and accelerating atomic diffusion. It should be noted that owing to the limitations in detection by XRD, the possible formation of tiny amount of titanium oxide phase during composites production could not be investigated.

### 3.3. Microstructure Characterization by FESEM

**Figure 4:**
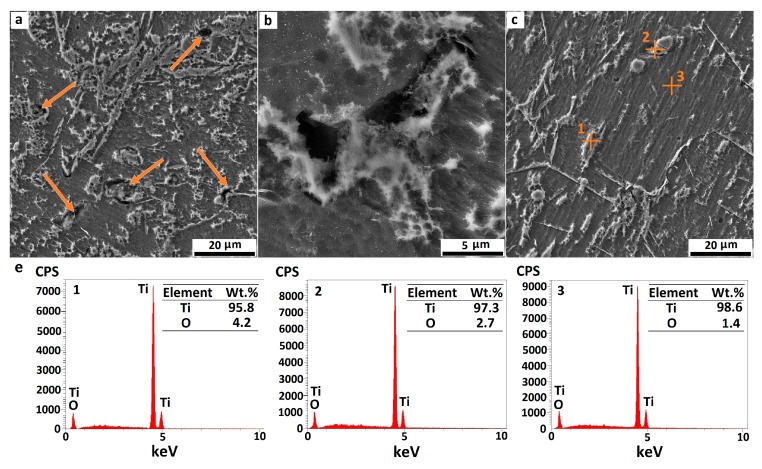
(**a**) SEM image of pure titanium composite sintered for 1 h (pores are shown with arrows), (**b**) high magnification image of a pore in pure titanium composite sintered for 1 h, (**c**) SEM image of pure titanium composite sintered for 5 h, and (**d**) energy-dispersive detector (EDS) analysis of selected points in (images 1, 2 and 3 represent the EDS analysis of points 1, 2 and 3 in (c), respectively).

A FESEM was used to investigate the microstructure and surface morphology of pure Ti and Ti/GNS composites. Figure 4 depicts the influence of sintering time on the surface morphology of pure Ti. Comparing the surface morphology of a pure Ti sample sintered for 1 h with the sample sintered for 5 h reveals that with increasing the sintering time at 1273 K, the structure of the composite has become more homogenous, which is attributed to the coherence between the composite particles, and the reduction of porosity by filling the gaps between the pores. It is also clear that the surface of pure Ti samples is almost smooth and free of macrostructural defects, especially in a specimen sintered for 5 h. The EDS analyses of selected points in Figure 4c are illustrated in Figure 4d. As can be seen, although the milling and sintering processes were carried out in an argon-protected vacuum environment, in addition to the detection of titanium, a slight amount of oxygen was found is some points as a result of the surface oxidation of powders due to the high reactivity of titanium. The oxygen confirms the presence of titanium oxides in the specimens.

**Figure 5:**
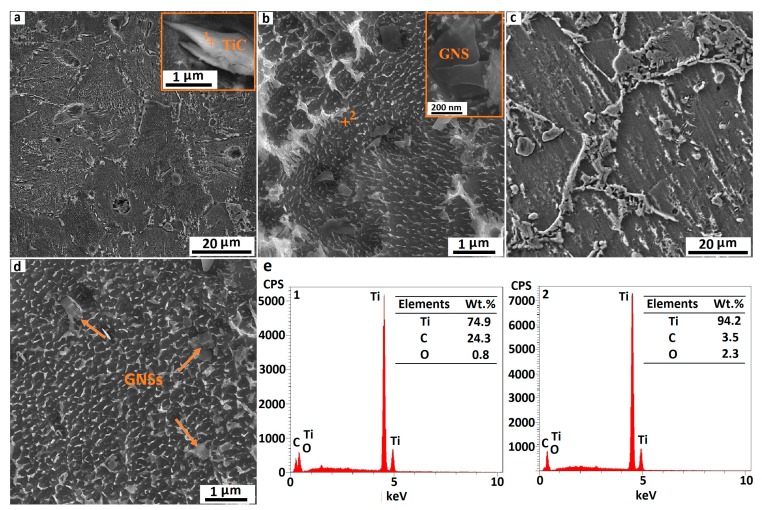
(**a**,**b**) SEM image of Ti/0.5GNSs composite sintered for 1 h, (**c**,**d**) SEM image of Ti/0.5GNSs composite sintered for 5 h, and (**e**) EDS analysis of selected points (images 1 and 2 represent the EDS analysis of points 1 and 2 in (**a,b**), respectively).

Figure 5 depicts the SEM microstructure of the composites with 0.5 wt. % of GNSs sintered at 1273 K for 1 and 5 h. As was predicted, GNSs were reacted with the matrix and TiC particles were formed. Figure 5a shows that the TiC particles were formed inside the grains as well as along the grain boundaries, with different sizes up to 3 μm. The formation of TiC particles on the grain boundaries of titanium powders limited grain growth. Accordingly, the maximum grain size of the composite containing 0.5 wt. % of GNSs was less than 20 μm. The shape and quality of the bonding of a TiC particle with a titanium matrix in the Ti-0.5G-1 sample is shown in Figure 5a. A strong bond between the TiC particles and titanium matrix was obtained by local reaction, which enhanced the mechanical properties [39,40]. According to Figure 5b, some nonreactive, thin, and small GNSs were observed in the Ti-0.5G-1 specimen, indicating that the GNSs were not completely reacted with titanium within 1 h of sintering at 1273 K. Therefore, it is worth noting that these very thin GNSs could also impede the grain growth of the matrix significantly because of their high specific surface area.

A comparison of Figure 5a,c reveals that by increasing the sintering time, the volume fraction of the TiC phase increased and the density of the composites enhanced due to the removal of the cavities. The reaction between titanium and GNSs increased by the increment of the sintering time and, consequently, higher amounts of TiC were formed. On the other hand, by increasing the time of sintering, the shape of the TiC particles was turned from a stretched and disk shape in the Ti-0.5G-1 sample to homogeneous and massive particles in the Ti-0.5G-5 sample. Moreover, the size of TiC particles increased to 6 μm after 5 h of sintering. The extra formation of TiC in the Ti-0.5G-5 specimen could impede the growth of grains. Therefore, despite increasing the time of sintering, the grain size of the composite decreased slightly. It should also be noted that a smaller quantity of unreacted GNSs was present in the microstructure of composite sintered for 5 h (Figure 5d) compared to that of the composite sintered for 1 h. According to the EDS graph of composites containing 0.5 wt. % of GNSs in Figure 5e, in addition to the presence of titanium and a slight amount of oxygen, a reasonable amount of carbon was also present. The EDS spectra of point 1 in Figure 5a show the presence of a high amount of carbon, which confirms the formation of TiC in the microstructure.

**Figure 6:**
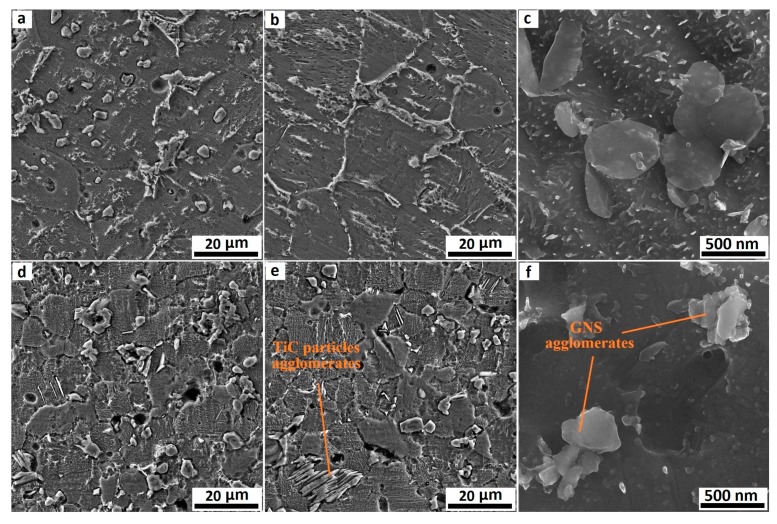
SEM image of Ti/GNS composite containing 1 and 1.5 wt. % of GNSs, (**a**) Ti/1GNS composite sintered for 1 h, (**b**,**c**) Ti/1GNSs composite sintered for 5 h, (**d**) Ti/1.5GNSs composite sintered for 1 h, and (**e**,**f**) Ti/1.5GNSs composite sintered for 5 h.

Figure 6 illustrates the SEM image of composites containing 1 and 1.5 wt. % GNS sintered at different times. A comparison of the SEM images in Figure 6 shows that an increment in the weight percentage of GNSs leads to the formation of a larger volume fraction of TiC. This increase is more obvious in composites sintered for a longer period. As can be seen, the TiC particles in the Ti/1GNSs composites possess a spherical shape irrespective of sintering time, while most of the particles have a disc-shape appearance in the Ti/0.5GNSs composite sintered for 1 h. It is also clear that the excessive increase of GNSs as a source of TiC formation caused the formation of the TiC agglomerates in the boundaries of the matrix. This reduced the homogeneous desperation of TiC particles in Ti/1.5GNSs composites.

Similar to what happened to Ti/0.5GNSs composites, increasing the time of sintering resulted in a more uniform distribution of TiC particles in the titanium matrix, as well as a reduction of porosity. Due to the agglomeration of GNSs, Ti/1.5GNSs composites contained a higher number of defects and porosity compared to the composites with a lower GNS content. In addition to the presence of TiC particles as reinforcing materials, there are some unreacted GNSs in the microstructure, as shown in Figure 6c. This can have positive effects on the mechanical properties of the composites. The theoretical analysis has indicated that the localized residual stresses in the vicinity of these nanofillers are large enough to generate dislocations that can release the remaining titanium tension and improve the composite strength [41,42]. According to the SEM image of the Ti/1.5GNSs composite in Figure 6f, as a result of increasing the GNSs content to 1.5 wt. %, the quantity of GNSs in the microstructure increased and, consequently, some GNS agglomerates were formed. Due to the high specific area of GNSs, the sheets formed agglomerates of higher width and thickness through overlapping. These agglomerates usually connect to cavities and porosities, giving rise to a reduced quality of connection bonds and, consequently, a weakening of the load transfer power and a deterioration of mechanical properties [43,44,45]. On the other hand, the presence of the remaining GNSs in the matrix and the formation of a larger fraction of TiC at the boundaries of the Ti/1.5GNSs composite impeded the grain growth of the matrix and decreased the grain size. It can be deduced that by increasing the weight percentage of GNSs to 1.5 wt. %, despite a reduction of the grain size, the mechanical properties of the composites decreased due to the formation of GNSs and TiC particle agglomerates.

### 3.4. Mechanical Properties

#### 3.4.1. Microhardness

**Figure 7:**
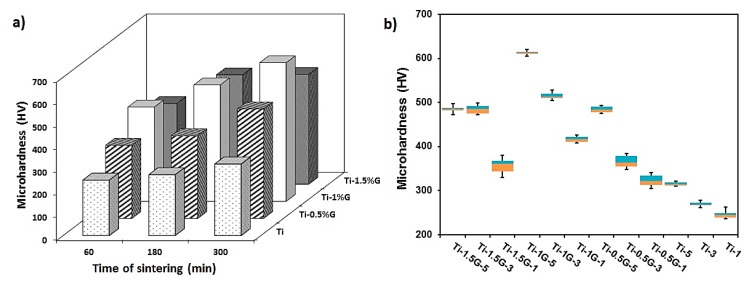
(**a**) 3-D column diagram of the effect of GNSs’ weight percentage on Vickers microhardness of titanium matrix composite sintered at different time periods, and (**b**) Box plot diagram of titanium/graphene composites.

In order to investigate the hardness uniformity as well as the influence of graphene content and sintering time on the hardness of the composites, the Vickers microhardness test was performed. Table 2 and Figure 7 display the microhardness values of sections specified in Figure 2. As shown in the 3-D column diagram in Figure 7a, the hardness of the composites increased significantly through the addition of GNSs to the titanium matrix. Increasing the GNSs content up to 1 wt. % led to an increment in the microhardness of the specimens, while increasing the GNS content more than 1 wt. %, caused a reduction in the microhardness. The microhardness results also imply that for composites with the same content of GNSs, higher hardness values are achieved for longer sintering times. The reasons may lie in the porosity removal and density increment, as well as the formation of the larger fraction of TiC reinforcements and improvement of uniform distribution of TiC particles in the matrix. It is observed that the composite containing 1 wt. % GNSs sintered for 5 h had the highest value of hardness (about 613 HV). This hardness was about twice the hardness of the carbon block/titanium composite produced by Thotsaphon et al. [46] and 52% higher than the titanium composite reinforced with carbon nanotubes produced by Vasanthakumar et al. [47]. This comparison indicates a higher effect of GNSs in improving the hardness of the titanium composite compared to those reinforcements.

Figure 7b shows the box plot diagram of various points of the samples surface. As can be seen, among pure titanium specimens, the Ti-5 sample has a more uniform microhardness than the other samples due to the longer sintering time. In addition, as illustrated in the box plot diagram, among the samples of titanium/graphene, the Ti-1G-5 sample possesses more uniform microhardness because of the proper sintering time and the optimum GNS content.

The hardness improvement of the composites as a result of GNS addition can be studied in several ways. As mentioned above, TiC particles were formed by the reaction of titanium with graphene and were distributed in the microstructure of the composites. These particles, as a secondary hard phase, create resistance to localized plastic deformation and consequently improve composite hardness. In addition, by increasing the formation of TiC particles, the grain growth of the matrix becomes limited, and accordingly, the reduction of grain size improves the composites’ hardness. Furthermore, the other reason for the hardness enhancement of the titanium/graphene composites is the presence of the remaining GNSs in the composite structure. Since graphene has extraordinary mechanical properties, during imposing the load to the composite, a large part of the load is taken by GNSs and, consequently, composite deformation is prevented [11]. On the other hand, similar to TiC particles, the presence of these nanosheets in the structure could control the grain growth of the matrix.

#### 3.4.2. Shear Stress

**Figure 8:**
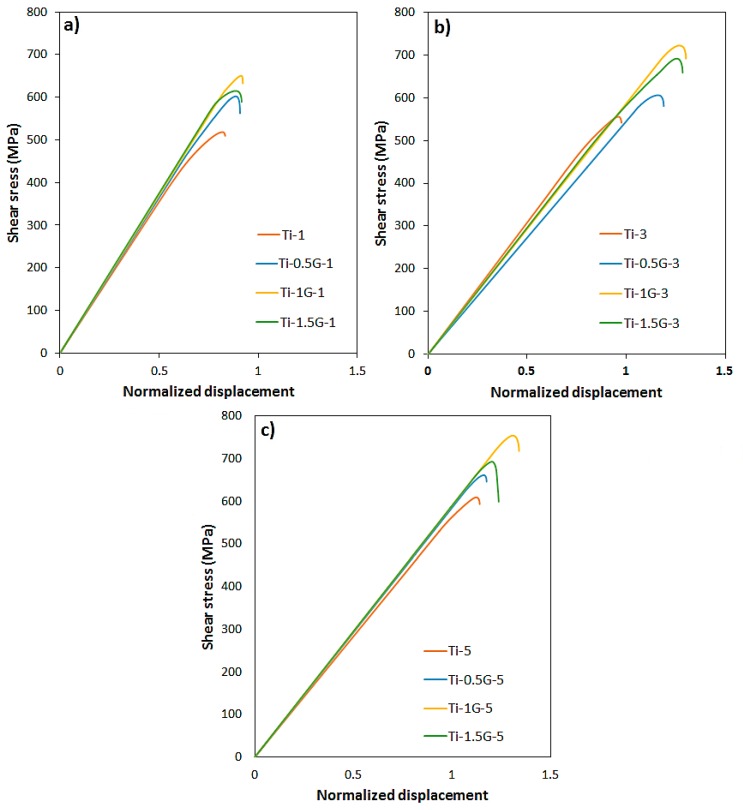
Shear stress versus normalized displacement curves of composites sintered at various time periods, (**a**) sintered for 1 h, (**b**) sintered for 3 h, and (**c**) sintered for 5 h.

**Figure 9:**
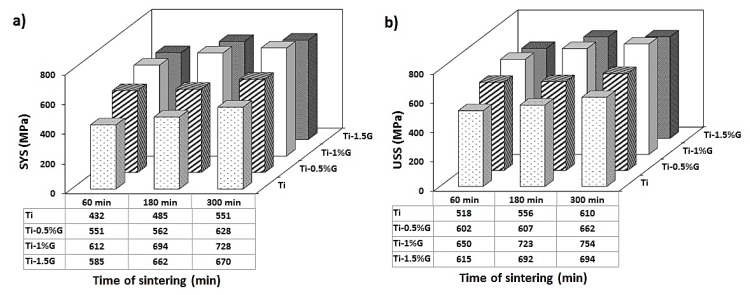
3-D column diagram of the effect of GNSs’ weight percentage on shear yield stress (SYS) (**a**) and ultimate shear stress (USS) (**b**) of titanium matrix composite sintered at different time periods.

The variation of computed shear stress versus normalized displacement is plotted in Figure 8. As is known, similar to the tensile stress–strain curve, in the shear punch diagram, after the elastic behavior, the curve deviates from linear state and continues to the maximum stress. The deflection point, obtained by drawing a tangent from the linear curve, is considered as the shear yield strength (SYS) and maximum stress is also indicated as the ultimate shear strength (USS). The values of SYS and USS of the samples are presented in Figure 9. It is obvious that by increasing the content of the GNSs up to 1 wt. %, the shear yield and ultimate shear strengths of the samples increased notably compared to those of pure titanium sample, especially in the Ti-1G-5 sample. An addition of 1 wt. % GNSs to pure titanium led to an increment of SYS and USS from 551 and 610 MPa to 728 and 754 MPa, respectively, in composites sintered for 5 h. According to Figure 9, a sintering time increment increased the samples SYS and USS. In fact, increasing sintering time promoted diffusion and reduced cavities and porosities; consequently, it increased the density of the samples significantly. Furthermore, a longer sintering time contributed to a greater production and more uniform distribution of TiC particles and unreacted GNSs in the matrix. It is worth mentioning that the SYS and USS of the Ti-1-5 sample were about 3 times higher than those of the rolled pure titanium, and even 22 and 46% higher than those of pure titanium processed by multidirectional forging (MDF) up to six passes, respectively [34], showing the positive role of GNSs in the improvement of the mechanical properties of titanium. In addition, the SYS and USS of the pure titanium sample sintered for 5 h were about 2.6 and 1.6 times more than those of the rolled pure titanium sample, respectively [34], which indicates the efficiency of powder metallurgy as an effective and simple method of production.

In general, two main reasons can be considered for increasing the strength of titanium/GNS composites: The formation of TiC particles during sintering and the presence of unreacted GNSs in the structure. The presence of TiC particles and its uniform distribution in the titanium matrix, in addition to limiting the grain growth of the matrix and prevention of the dislocation movement, hinders the deformation of the matrix during loading and carries out part of the external load as reinforcing materials [48,49,50]. At the same time, the presence of unreacted GNSs in the structure also increases the composite strength in several ways. The existence of these GNSs due to its two-dimensional structure and high surface area has a pinning effect that restricts the motion of grain boundary and refines the grain size [51]. Moreover, the strong bonding at the Ti/GNSs interface is very important in excellent load transfer from the matrix to GNSs, which is dependent upon interfacial bonding between the matrix and GNSs [52]. Orowan looping is also an important reason for improving the strength of composites [41,53,54]. According to theoretical analysis, the residual localized stresses around the GNSs in the structure create dislocation loops around the GNSs and will prevent the movement of dislocations and the propagation of cracks. In addition to the size of the reinforcements, their uniform distribution also plays an important role in this mechanism [42,55]. In fact, alongside the grain size and the quality of the load transfer, the strengthening of the composite depends on the density of the dislocations which are attributed to the surface area of reinforcements. Therefore, the presence of GNSs in the composites with a large surface area improves the strength of the composites by increasing the dislocations’ density. In addition to the aforementioned mechanisms, a thermal mismatch of components has been also reported as an effective mechanism for composite strengthening. Since the value of the thermal expansion coefficient (CTE) for GNSs is smaller than that for titanium, the presence of significant mismatch between these coefficients causes the prismatic punching of dislocations at the interface, and enhances the strength of composites [56,57].

As can be demonstrated in Figure 7 and Figure 9, by increasing GNS content more than 1 wt. %, the improvement of mechanical properties reduced reasonably. This was due to the excessive content of GNSs in the matrix, which caused the agglomeration of GNS and TiC particles, as shown in the SEM images. Owing to the high surface area of the GNSs, the nanosheets will form agglomerates with a higher width and thickness through overlapping. However, since graphene is a two-dimensional material and its inner sheets form a weak Van Der Waals bond in contrast to its outer sheets, these agglomerates, as a weak structure, are the cause for the formation of pores and porosity in the matrix and will reduce the improvement of the mechanical properties [43,44,45]. Moreover, the aforementioned weak bonds are the cause for the diminishing of load transfer power from the matrix to the reinforcement. On the other hand, the relatively weak bond between the layers of these agglomerated GNSs leads to the easier gap and slippage between their layers. Briefly, it can be concluded that the formation of these agglomerates has a negative effect on the mechanical properties of the composites, as a result of weakening the bond between GNSs and matrix, as well as creating imperfections and cavities.

## 4. Conclusions

Titanium/GNS composites were fabricated through cold pressing and sintering processes. The results demonstrated that in spite of the simplicity of this method, it is highly efficient to fabricate titanium/GNS composites with remarkably high mechanical properties. The main conclusions of the current investigation can be summarized as follows:

1. The density measurement by Archimedes’ method showed that by increasing sintering time, the density of the samples enhanced reasonably, while increasing the weight percentage of GNSs caused a slight reduction of density. Ti/GNS composites with a reasonable high density (more than 99.5% of theoretical density) were fabricated after sintering for 5 h.
2. XRD and SEM investigations confirmed the formation of TiC particles in the titanium/GNS composites due to the reaction of GNSs with the titanium matrix, which played an effective role in improving the mechanical properties of the composites. SEM images also revealed that an increase in the weight percentage of GNSs as well as sintering time resulted in the formation of a larger volume fraction of TiC particles. On the other hand, the outstanding unreacted GNSs remaining in the microstructure were also effective in the enhancement of mechanical properties of the composites.
3. A characterization of the microstructure by SEM demonstrated that the excessive content of GNSs in composites containing 1.5 wt. % GNSs resulted in the agglomeration of GNSs and TiC particles in the microstructure and consequently, the improvement of mechanical properties reduced drastically.
4. Mechanical properties experiments revealed that an increase in GNS content up to 1 wt. % and an increment of the sintering time caused an enhancement in the microhardness and shear strength of the composites. Microhardness, shear yield strength, and ultimate shear strength of the composite containing 1 wt. % GNSs sintered for 5 h which possessed the highest mechanical properties were 613 HV, 728 MPa, and 754 MPa, respectively.

## Figures and Tables

**Table 1:** Theoretical and experimental densities of composites containing various amounts of GNSs sintered at different times.

Samples	Time of Sintering (h)	Content of GNSs (wt. %)	Theoretical Density (g/cm^3^)	Experimental Density (g/cm^3^)	Percentage of Density (%)
**Ti-1**	1	0	4.506	4.382	97.2
**Ti-3**	3	0	4.506	4.410	97.8
**Ti-5**	5	0	4.506	4.476	99.3
**Ti-0.5G-1**	1	0.5	4.480	4.345	97.0
**Ti-0.5G-3**	3	0.5	4.480	4.395	98.1
**Ti-0.5G-5**	5	0.5	4.480	4.467	99.7
**Ti-1G-1**	1	1	4.450	4.337	97.5
**Ti-1G-3**	3	1	4.450	4.388	98.6
**Ti-1G-5**	5	1	4.450	4.440	99.7
**Ti-1.5G-1**	1	1.5	4.420	4.275	96.7
**Ti-1.5G-3**	3	1.5	4.420	4.301	97.3
**Ti-1.5G-5**	5	1.5	4.420	4.334	98.1

**Table 2:** The Results of the Vickers microhardness test for the specified sections in composites produced in different conditions.

Sample	Vickers Microhardness								
	A_1_B_1_	A_1_B_2_	A_1_B_3_	A_1_B_4_	A_2_B_1_	A_2_B_2_	A_2_B_3_	A_2_B_4_	Average
**Ti-1**	244	237	252	263	239	245	247	236	245
**Ti-3**	271	268	266	275	278	268	269	259	269
**Ti-5**	320	322	316	312	319	310	312	314	316
**Ti-0.5G-1**	332	311	338	341	314	305	328	315	323
**Ti-0.5G-3**	384	350	378	348	380	356	357	371	366
**Ti-0.5G-5**	479	485	493	494	475	489	483	475	484
**Ti-1G-1**	427	423	412	408	413	419	421	410	417
**Ti-1G-3**	514	520	509	505	528	512	511	521	515
**Ti-1G-5**	615	611	609	620	612	617	614	606	613
**Ti-1.5G-1**	330	359	338	372	366	345	380	363	357
**Ti-1.5G-3**	476	486	492	499	472	492	483	473	484
**Ti-1.5G-5**	473	484	486	503	479	494	485	483	486
